# Comparative Genomics of *Serratia* spp.: Two Paths towards Endosymbiotic Life

**DOI:** 10.1371/journal.pone.0047274

**Published:** 2012-10-15

**Authors:** Alejandro Manzano-Marín, Araceli Lamelas, Andrés Moya, Amparo Latorre

**Affiliations:** 1 Institut Cavanilles de Biodiversitat i Biologia Evolutiva, Universitat de València, Valencia, Spain; 2 Unidad Mixta de Investigación en Genómica y Salud del Centro Superior de Investigación en Salud Pública (Generalitat Valenciana) y del Institut Cavanilles de Biodiversitat i Biologia Evolutiva de la Universitat de València, Valencia, Spain; University of Vienna, Austria

## Abstract

Symbiosis is a widespread phenomenon in nature, in which insects show a great number of these associations. *Buchnera aphidicola*, the obligate endosymbiont of aphids, coexists in some species with another intracellular bacterium, *Serratia symbiotica*. Of particular interest is the case of the cedar aphid *Cinara cedri*, where *B. aphidicola* BCc and *S. symbiotica* SCc need each other to fulfil their symbiotic role with the insect. Moreover, various features seem to indicate that *S. symbiotica* SCc is closer to an obligate endosymbiont than to other facultative *S. symbiotica*, such as the one described for the aphid *Acirthosyphon pisum* (*S. symbiotica* SAp). This work is based on the comparative genomics of five strains of *Serratia*, three free-living and two endosymbiotic ones (one facultative and one obligate) which should allow us to dissect the genome reduction taking place in the adaptive process to an intracellular life-style. Using a pan-genome approach, we have identified shared and strain-specific genes from both endosymbiotic strains and gained insight into the different genetic reduction both *S. symbiotica* have undergone. We have identified both retained and reduced functional categories in *S. symbiotica* compared to the Free-Living *Serratia* (FLS) that seem to be related with its endosymbiotic role in their specific host-symbiont systems. By means of a phylogenomic reconstruction we have solved the position of both endosymbionts with confidence, established the probable insect-pathogen origin of the symbiotic clade as well as the high amino-acid substitution rate in *S. symbiotica* SCc. Finally, we were able to quantify the minimal number of rearrangements suffered in the endosymbiotic lineages and reconstruct a minimal rearrangement phylogeny. All these findings provide important evidence for the existence of at least two distinctive *S. symbiotica* lineages that are characterized by different rearrangements, gene content, genome size and branch lengths.

## Introduction

Symbiosis is a widespread phenomenon among all branches of life. Especially, insects show a tight relationship with a variety of these organisms [1] mostly having a metabolic foundation, as bacteria provide the insects with the nutrients lacking in their diet. This is the case for many aphids that maintain a close association with the ancient obligate bacterium *B. aphidicola*. The association is mutualistic as none of the partners can subsist without the other one. The aphid gives *B. aphidicola* a stable environment and in return this gives the aphid the nutrients lacking from its diet, the plant’s phloem. At present the genomes of *B. aphidicola* from seven aphid species have been sequenced [2], [3], [4], [5], [6], [7], [8], [9], with the smallest genome found in the aphid *C. cedri* (*B. aphidicola* BCc), with a genome size of 416 Kb coding solely 357 protein-coding genes. This contrasts with other less genomically reduced *Buchnera*, like the one from the aphid *A. pisum* (*B. aphidicola* BAp). The symbiotic role of *B. aphidicola* BCc has even been questioned since, contrary to other *Buchnera*, it was found unable to fulfil some of its symbiotic functions [5]. In addition to *B. aphidicola*, some aphids harbour other endosymbiotic bacteria called secondary or facultative endosymbionts, such as *Hamiltonella defensa* [10], *Regiella insecticola* [11] and *S. symbiotica* [12], [13], whose genomes have recently been sequenced. Although primarily transmitted vertically, these facultative bacteria undergo occasional horizontal transfer [14], [15], [16], [17]. These three bacteria have been shown to benefit the host, providing defense against fungal pathogens, parasitoid wasps or even increasing survival after environmental heat stress (revised in [17]). However, as they are facultative, they do not seem to be essential to the insect’s survival. An interesting genomic feature from these young associations, contrary to more ancient ones, is the massive presence of mobile genetic elements in their genomes [18], [19], [20], [21], which would cause their genomes to undergo a number of rearrangements as compared to their free-living relatives.

Species of the genus *Serratia* have been found in numerous places such as water, soil, plants, humans and invertebrates like many insects [22]. The presence of *Serratia* in insects digestive tract has been speculated to be of plant origin, since the hemolymph cannot prevent the multiplication of potential pathogens [23]. On the other hand, *S. symbiotica* is one of the most common facultative symbionts in many aphids. In *A. pisum*, it has been found to confer defense against environmental heat stress [24], [25], [26], [27]. In a study into the evolution of *S. symbiotica* endosymbionts, both phylogenetic and morphological evidence was found of the possible existence of at least two different *S. symbiotica* clades named A and B [28]. Clade A shows characteristics resembling a facultative symbiont, whereas clade B resembles more to an obligate-like endosymbiont [28], [29]. The genome sequencing of *S. symbiotica* from *A. pisum* (*S. symbiotica* SAp) [12] and *C. cedri* (*S. symbiotica* SCc) [13], belonging to clade A and B respectively, has revealed very different genomic features (see **Table 1**). *S. symbiotica* SAp possesses a genome size of around half of that of FLS, over two thousand fewer protein coding genes and an impressive extent of pseudogenes (550), giving some indication of a relatively recent inactivation of many genes. On the other hand, *S. symbiotica* SCc is immediately striking in that it presents a genome size around 1 Mb smaller than that of *S. symbiotica* SAp, a reduced set of protein coding genes, a very low coding density and GC content and surprisingly depleted of mobile genetic elements [13] identified in other recently derived endosymbiotic relationships [10], [11], [19], including *S. symbiotica* SAp [12].

**Table 1:** Species, accession numbers and genomic features comparison of *Serratia* spp. and selected *B. aphidicola* genomes.

Strain	Accession	Genome size (Mb)	GC%	CDS	Host	lifestyle
***S. odorifera*** ** 4R×13**	ADBX00000000 (WGS)	5.36	56	4668	*Brassica napus*	Free-living
***S. proteamaculans*** ** 568**	CP000826, CP000827	5.45	55	4891+51	*Populus trichocarpa*	Free-living
***S. marcescens*** ** Db11**	http://www.sanger.ac.uk/resources/downloads/bacteria/serratia-marcescens.html	5.11	59	4763	*Drosophila melanogaster*	Free-living
***S. symbiotica*** ** SAp**	AENX00000000 (WGS)	2.79	52	2098	*A. Pisum*	Falcultativeendosymbiont
***S. symbiotica*** ** SCc**	CP000826	1.76	29	672	*C. Cedri*	Obligateendosymbiont
***B. aphidicola*** ** BCc**	CP000263, AY438025, EU660486	0.42	20	357+5	*C. cedri*	Obligateendosymbiont
***B. aphidicola*** ** BAp APS**	BA000003, AP001071, AP001070	0.64	26	564+10	*A. pisum*	Obligateendosymbiont

Genomic features for FLS and both *S. symbiotica* along with their *B. aphidicola* partners, evidencing each *S. symbiotica* genomoic reduction compared to their free-living relatives.

The pan-genome approach for studying evolutionary relationships at a certain taxonomical level has been proved a very powerful tool to study diverse aspects of genomic, functional and structural characteristics of groups of genomes [30], [31], [32], [33], [34]. The term “pan-genome” has been used to refer to the collection of the core genome (genes shared by all strains and probably encoding fundamental functions of the biology and phenotype of the species) and an accessory genome (constituted from the genes present in some but not all strains) [35], this latter one including genes that are essential for a certain environmental adaptation [33] and linked to capsular serotype, virulence, adaptation and antibiotic resistance probably giving some indication to the organisms lifestyle [36].

In the present study, due to the findings in both *S. symbiotica* genomes sequenced so far, we wanted to study the diverse processes that occurred once these organisms adapted to an intracellular environment. These include their genetic reduction, rearrangements, and also how the current functional state of their respective *B. aphidicola* partner explains the current functionality of each *S. symbiotica*. It is worth mentioning that we have a unique opportunity with the genus *Serratia* provided by the availability of complete genomic data from three different snapshots distributed throughout the transition from free-living (*Serratia proteamaculans* 568, *Serratia marcescens* Db11 [37] and *Serratia odorifera* 4R×13 [38]) passing through facultative endosymbiosis (*S. symbiotica* SAp) to obligate endosymbiosis (*S. symbiotica* SCc).

In order to gain insight into the level of genome reduction undergone by the two *S. symbiotica* strains, we first defined a pan-genome for the genus *Serratia* using the annotated CDSs for the five strains mentioned above. We then went on to explore specific subspaces of the pan-genome, such as some of the genes retained outside the core genome and strain-specific genes of each *S. symbiotica*. We found a massive level of genomic reduction in *S. symbiotica* SCc, even when compared to *S. symbiotica* SAp in which a great number of accessory genes are still retained in its genome. The differential genetic reduction suffered by these endosymbionts also became evident, finding a number of CDSs shared with other *Serratia* but not between them. We then went on to analyze and compare the functional profiles for each *Serratia* strain used to reconstruct the pan-genome along with the pan-genome itself and the core-genome using the Cluster of Orthologous Groups (COG) functional categories [39]. We were interested in observing the functional clustering and shifting of both endosymbiotic strains compared to the FLS. We also compared each of the *S. symbiotica* functional profiles to that of the average of FLS in order to detect profile modification of individual categories to be able better understand the functional constraints under which each *S. symbiotica* genome has evolved and the divergence of these endosymbiotic strains. We observed that the functional profile of *S. symbiotica* SCc clustered very close to that of the core-genome, supporting a very advanced stage of genetic reduction. In addition, we wanted to analyze the process of genome rearrangements and genetic evolution that these endosymbionts have undergone. To do so, we first defined a set of single-copy shared genes which were taken as a base to study the different arrangements of these among the different *Serratia* genomes and to perform a phylogenetic reconstruction of the *Serratia* spp. In contrast to the perfect conservation of the single-copy shared genes order and orientation in the FLS, we found a great level of reordering even between the two endosymbiotic *Serratia* strains. Also, we quantified the minimal rearrangements needed to get to an ancestral gene order through a minimal number of rearrangements tree. Finally, the phylogenetic analysis confidently resolved the relationships among the different *Serratia* strains used in this study, allowing us to propose a probable origin for the endosymbiotic lineages.

## Results and Discussion

### Pan-genome’s General Features

**Figure 1:**
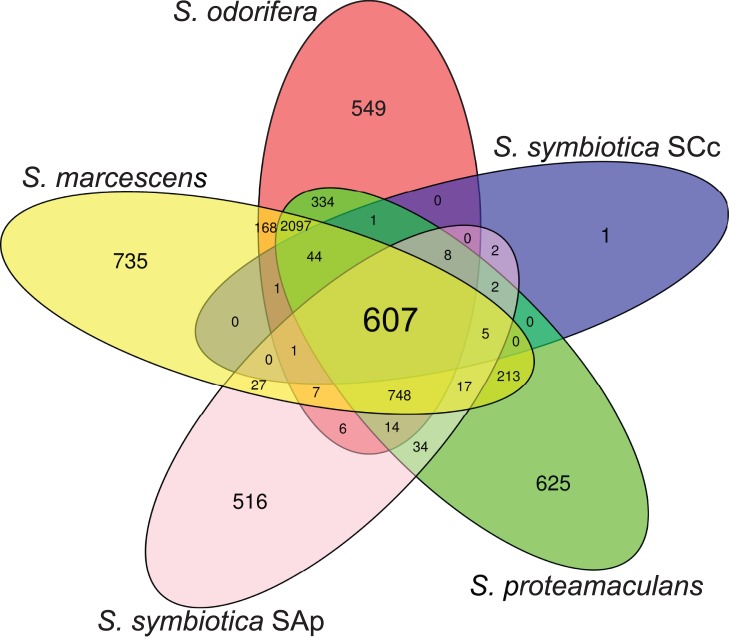
Pan-genome of *Serratia* spp. Euler diagram displaying the number of clusters found on each subspace of the pan-genome. The pan-genome defined here as the total collection of CDS clusters found in *S. symbiotica* SCc, *S. symbiotica* SAp, *S. proteamaculans* 568, *S. odorifera* 4R×13 and *S. marcescens* Db11 (first two obligate and facultative endosymbiont respectively and the rest free-living).

To gain insight into the process of reductive evolution undergone by the adaptation from a free-living state to an endosymbiotic-lifestyle, we reconstructed a pan-genome for the genus *Serratia*. It is worth mentioning that, at present and to our knowledge, it is the only bacterial genus for which full genome sequences for both endosymbiotic and free-living species are available. General features for each strain, together with the ones from the two *Buchnera* strains sharing their host with *Serratia* in the same aphid (*B. aphidicola* BAp and BCc), are summarized in **Table 1**. The CDSs of the two available endosymbiotic strains (the facultative *S. symbiotica* SAp and the obligate *S. symbiotica* SCc) and three free-living ones (*S. marcescens*, *S. proteamaculans* and *S. odorifera*) were recovered from their respective sources. After a clustering of the organisms’ protein sequences we ended up with 4, 469 orthologous clusters of proteins, leaving 2, 293 unclustered proteins, corresponding to the strain-specific genes. To visualize the clusters’ location within the pan-genome subspaces, an Euler diagram was computed (**Figure 1**). The first remarkable feature is finding very few clusters (607) from the pan-genome in the core (8.98%). While, if we also take into account the genes shared by the three FLS plus the core (3, 452), the percentage is greatly increased (51.05%) due to the presence of the endosymbiotic genomes, mainly the genome from *S. symbiotica* SCc, which displays an extensive genomic reduction due to its adaptation to an intra-cellular lifestyle [13]. Regarding the strain-specific genes, almost half of them (47.45%) are hypothetical proteins and 7.07% are putative ones. This is not surprising since it has been described that most of the strain specific genes in a pan-genome are hypothetical genes, genes which may be product of over-annotation given their generally reduced sizes, or ORFan genes [40].

It is worth mentioning that all the coding genes present in the annotated CDSs of *S. symbiotica* SCc are present one gene per cluster, showing no evidence of genetic redundancy and supporting its extreme reductive process compared to the other *S. symbiotica*. This is important since taking into account that the levels of duplication of the other *Serratia* are higher (*S. marcescens* 3.6% duplicated genes, *S. odorifera* 3.2%, *S. proteamaculans* 6.6% and *S. symbiotica* SAp 3.7%). In addition, almost all of the coding genes from *S. symbiotica* SCc (607 out of 672) clustered into the core. Not surprising for obligate endosymbionts since the reductive process tends to reduce both redundancy and genetic repertoire, conserving the genes that allow the bacteria to sustain themselves and fulfil their role in the symbiotic association.

### *S. symbiotica* Strain-specific Genes

Amazingly, there is only one strain-specific gene for *S. symbiotica* SCc. A 67 amino-acid hypothetical protein, which on a BLAST search against nr was found to vaguely resemble (less than 56% covered and 62% identity) another hypothetical protein in *S. marcescens* (genbank locus tag HMPREF0758). This displays the massive genetic decay that *S. symbiotica* SCc has suffered, basically losing all its strain-specific genes, contrary to *S. symbiotica* SAp, which still retains many of them (516 gene clusters), reminding us of characteristics of free-living *Serratia*. Mostly, the genes present in these clusters code for phage proteins (26), transposases (29) or are annotated as hypothetical proteins (304), while the rest are annotated mostly as putative proteins (157, related to conjugative systems, pili, fimbria, transporters and some others). Due to the accessory nature of these groups, they might eventually be degraded in the genome reduction process if this endosymbiont continues to accommodate itself in the system.

### *S. symbiotica* Genes Outside the Core

Two genes are shared by both *S. symbiotica*, *epsI* and *rfaI*, coding for a glycosyl transferase and a lipopolysaccharide 1,3-galactosyltransferase respectively. The two are involved in cell envelope biogenesis (outer membrane), which could explain the reason why these do not cluster with other members coming from FLS. These type of proteins have been found to show weak signals of incongruence, due to being genes involved in diversifying selection, coding for antigenic proteins exposed at the cell surface [41].

Interesting are the clusters shared by both endosymbiotic bacteria (*S. symbiotica* SAp and SCc) and FLS regarding fimbrial genes. With *S. proteamaculans*, they shared the genes *fimA* and *pagO*, coding for the filament protein FimA involved in fimbrinbrial formation and a putative membrane protein respectively, and with *S. odorifera* and *S. proteamaculans* the gene *etfD* which codes for a protein associated to fimbrin. In both *S. symbiotica*, these fimbrial genes have been retained although at least in *S. symbiotica* SCc there is a loss of the capacity to form fimbrins. This probably means that this intersection is disappearing due to the deterioration of this pathway in the intracellular adaptation process, although it is also possible that it plays a role in the pathogen-host cross-talk or in infection. Other interesting genes are the two shared with *S. marcescens* and *S. proteamaculans* (*hha* coding for a haemolysin expression-modulating protein and *feoB* coding for a part of the iron transport system which makes an important contribution to its supply to the cell under anaerobic conditions), and one (*yidD*) shared with both *S. odorifera* and *S. proteamaculans*, which product clusters with a hemolysin from *S. proteamaculans*. Some hemolysins have been shown to allow bacteria to evade the immune system by escaping from phagosomes [42], and they are reported to serve as a way of obtaining nutrients from host cells. For example, in other organisms they have been involved in the iron uptake by pathogenic bacteria from their eukaryotic hosts [43].

Regarding the genes shared exclusively by *S. symbiotica* SCc and other free-living relatives, we found 44 clusters shared with *S. odorifera* and *S. marcescens*. These would be genes that reflect the differential genetic degradation between both *S. symbiotica*. In fact 24 (*bioA, bioB, cysJ, cysU, fruA, fruB, fruK, gyrA, hemC, hemD, mdtK, mrcA, nudF, pdxY, pnuC, queA, rseP, rsmB, thiK, thiP, yceB, yceG, yeiH, yhdP*) out of the 44 are present in *S. symbiotica* SAp as pseudogenes, 17 are completely absent (*ansP, apaG, cysC, cysD, cysG1, cysN, glnH, glnP, glnQ, lpp1, mltE, pyrC, queD, sufE, ybjN, ygdQ* and a hypothetical protein), and three (*apaH, uvrA, uvrC*) are annotated as genes with interrupting gaps; thus they were excluded from the analysis. Most of these genes are involved in the biosynthesis of cofactors like biotin, thiamine and haemoglobin, in electron transport chain and sugar transport, in agreement with [13]. The afore-cited study explains that genes involved in the categories of, for example sugar transport, electron transport chain and synthesis of some cofactors are affected by the genomic reduction process.

### Functional Relatedness and Divergence in *S. symbiotica*

**Figure 2:**
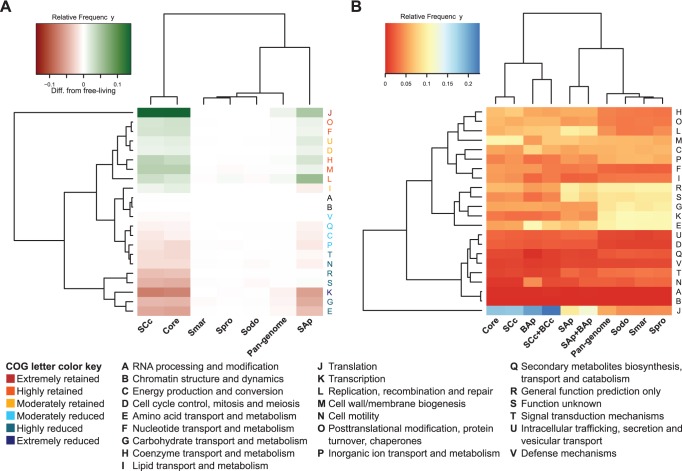
Functional profiles of core, pan-genome and selected *Serratia* and corresponding *Buchnera* genomes. **A.** Heatmap showing the two-way clustering of the COG profiles frequency differences from the FLS average. **B.** Heatmap showing the COG profiles from the selected *Serratia* and *Buchnera* genomes. On the right side of each heatmap, COG assignments for each row are displayed. In the bottom left, color key for the COG categories for the first heatmap in relation to the comparison *S. symbiotica* SCc vs FLS. In the bottom right, COG categories key. **BAp**:* B. aphidicola* from *A. pisum*; **BCc**: *B. aphidicola* from *C. cedri*; **Smar**: *S. marcescens* Db11; **Sodo**: *S. odorifera* 4R×13; **Spro**: *S. proteamaculans* 568; **SAp**:* S. symbiotica* from *A. pisum*; **SCc**: *S. symbiotica* from *C. cedri*.

To inquire into the functional roles of the selected *Serratia* strains, we assigned COG categories to each organism’s CDSs. Through a Kruskal-Wallis test on the absolute COG frequencies per organism we found significant differences in the core/pan-genome/*Serratia* COG profiles (χ^2^ = 72.84, df = 6, p-value = 1.07e-13). Through the same test using only the FLS, we found that they did not showed any significant difference (χ^2^ = 0.11, df = 2, p-value = 0.95). This indicates that the general functional composition of the FLS is highly conserved; being able to assume that any significant deviation from this profile in the endosymbiotic *Serratia* would be due to their adaptation to a new lifestyle. Then, to identify retained and reduced functional categories against FLS, as a way to asses functional divergence from FLS for each endosymbiotic *Serratia*, we mapped the COG profile differences from the FLS COG profile in a heatmap for the core, pan-genome and the individual genomes of *Serratia* (**Figure 2A**). As shown, the COG profile heatmap revealed a tight clustering of *S. symbiotica* SCc with the core-genome, expected from the fact of its gene content being too close to that of the core and giving support to its distinction from its facultative relative, *S. symbiotica* SAp, which remained as a separate group, probably exemplifying the functional profile of a facultative-symbiont lineage of *Serratia*.

Since *S. symbiotica* SCc shows the most extreme COG profile modification against FLS, we decided to take its functional profile to compare against the FLS average and afterwards check for the state of the same functional category in *S. symbiotica* SAp (**Table 2**; **Figure 2A**). We divided the results in the following categories (see Methods): **(i) Extremely retained**. Category **J**, meaning a great part of its gene repertoire is dedicated to basic functions for its cellular life maintenance as previously shown [44]. Also, most the universally conserved COGs fall into this category [45]. It is important to note that there is also an increase in this category in *S. symbiotica* SAp compared to FLS. **(ii) Highly retained**. Category **O**, as in the previous case this is not surprising, since it is normal that the genomic reductive process affects many of the genes involved in DNA repair, by which some proteins involved in post translational modification are common and which avoid missfolding or accumulation of defective peptides. This group includes the genes *groES* and *groEL*, that code for chaperone GroEL, which might mitigate the damage of reduced protein stability by maintaining a high cytoplasmic level of it in *B. aphidicola* [46], [47]. Category **F**, since *S. symbiotica* SCc, contrary to *B. aphidicola* BCc, still preserves the capacity to synthesize pyrimidines, and in the case of purines it could be recycling the aphids nucleosides to produce nitrogenous bases, complementary to the case of *S. symbiotica* SAp [12], [13]. Category **H**, showing the specialization of *S. symbiotica* SCc as a cofactor supplier [13]. Category **M**, explained by the noted ability of *S. symbiotica* SCc to still synthesize its own cell membrane, in contrast to the obligate endosymbiont *B. aphidicola* BCc which has lost many of the genes necessary for this function [5], [48]. On the other hand, *S. symbiotica* SAp still resembles the FLS in this category more closely. Category **L**, where previously shown that in spite of having a reduced number of genes compared to FLS, it still maintains those necessary for its genome replication [13]. Also the repair system (based on *E. coli*) by base excision is conserved, while the repair by recombination system is almost complete (*recB, recC, recD, sbcB, priB*) but missing the *recA* gene, as happens with the obligate endosymbiont *B. aphidicola* [13]. On the other hand, *S. symbiotica* SAp reveals more genes in this category compared to *S. symbiotica* SCc, as expected due to its less degraded genome. **(iii) Moderately retained**. Category **U**, mainly showing protein translocation and export related genes. This comes as no surprise, since in both cases, they had to adapt to import and export a variety of components due to the gene loss undergone in the adaptation process to intracellular life, which would require the conservation of many genes in this category. Category **D**, consisting among other things of the *fts* genes (*ftsL, ftsW, ftsA, ftsZ, ftsK*), cell-wall topological and structural coding genes (*mrdB, mreB*) and other cell cycle related proteins coding genes (*gidA, minC,minE, minD, ygbQ*). Category **I**, since *S. symbiotica* SCc, being not as advanced in genetic degradation as its partner *B. aphidicola* BCc, still preserves higher number of genes in this category, as also happens with *S. symbiotica* SAp which presents a greater repertoire, despite the relatively lower number of genes in the present category compared with FLS. **(iv) Moderately reduced** (which interestingly do not vary much between endosymbiotic *Serratia*). Category **V,** which in spite of showing almost no relative change in comparison to the FLS, there is a drastic decrease in absolute gene number against these. In *S. symbiotica* SCc these genes comprise only two genes involved in lipid transport (*msbA* and *lolD*), two multidrug efflux system genes (*mdtK* and *emrA*) and two predicted transporter subunits (*yadH* and *yadG*). Categories **Q** and **C**, comprising genes mainly involved in cellular life maintenance. Category **P**, where many transporters have been lost in *S. symbiotica* lineages, retaining only a limited repertoire. **(v) Highly reduced**. Category **T**, in which a massive loss of transcription regulators and sensor proteins has occurred. Category **N**, in which we see a vast reduction in absolute gene number from FLS. In this category the losses are mainly from flagellar proteins, fimbrial, pili and chemotaxis related proteins along with some outer membrane proteins. The reduction in both **T** and **N** categories can be explained by the stable environment in which the bacterial cell now resides, making many of the sensory systems and the motility mechanisms dispensable. Category **R** and **S**, displaying the different state of the genetic degradation of mainly strain-specific genes, since it has been noted that these are rich in proteins of unknown function [33], [40], which would explain why *S. symbiotica* SAp shows a pattern that is more similar to that of FLS. Category **G**, which in spite of the losses, is still able to import sugars from the aphid host (fructose), while *S. symbiotica* SAp can still use more (glucose, manose, etc.) [12]. Category **E**, where we find a common reduction in both *S. symbiotica* from FLS. This feature displays both endosymbionts reliance on *Buchnera* to supply many essential amino-acids partially or entirely [12], [13]. **(vi) Extremely reduced**. Here we find category **K**, where both *S. symbiotica* strains have lost a massive amount of transcriptional regulators. This also displays the loss of transcriptional regulation and responsiveness of reduced genomes of endosymbionts [49].

**Table 2:** *S. symbiotica* COG profile modification from FLS and between them.

	COG	SCc/FLS	SCc/Sap
**Extremely retained**	**J**	4.14	1.95
**Highly retained**	**O**	1.65	1.20
	**F**	2.03	1.40
	**H**	2.14	1.25
	**M**	1.67	1.39
	**L**	1.78	0.68
**Moderately retained**	**U**	2.36	0.86
	**D**	2.41	1.04
	**I**	1.30	1.88
**Moderately reduced**	**V**	0.72	0.73
	**Q**	0.58	1.03
	**C**	0.78	0.93
	**P**	0.74	0.95
**Highly reduced**	**T**	0.36	0.44
	**R**	0.62	0.63
	**S**	0.50	0.48
	**G**	0.57	1.14
	**E**	0.52	0.81
**Extremely reduced**	**K**	0.27	0.58

Ratios of relative number of genes for comparison of COG functional profile modifications present in the genomes of both *S. symbiotica*.

### Functional Convergence of *C. cedri* Bacterial Endosymbiotic Consortia and a Less Genomically Reduced *Buchnera*

Besides observing the specific categories in which both *S. symbiotica* find themselves altered compared to FLS, it is of importance to determine the evolution and fate of the two different associations each bacterium has established with *Buchnera*. It has been proposed that in *C. cedri* the bacterial consortium is involved in a co-obligate endosymbiosis, both members being required for the survival of the three partners in the system [13], while in *A. pisum Buchnera* alone is able to sustain the nutritional requirements of the aphid, without the need an extra member. Then, we decided to analyze whether the bacterial consortium in *C. cedri* could, at least generally, functionally resemble *B.aphidicola* BAp. To do so, we added the functional profiles from the corresponding *B. aphidicola* to those of its corresponding *S. symbiotica* partner (partner defined as the bacteria that share the same host) and performed a two-way clustering of the relative number of genes in each COG category using a heatmap (**Figure 2B**). First, the sum of *Serratia* and *Buchnera* in *C. cedri* clusters closer to the functional profile of *B. aphidicola* BAp than to any other *Serratia*, and also brings the functional profile of *B. aphidicola* BCc closer to other less genomically reduced *Buchnera* (See **Figure S1**). This data provides evidence that genetic decay in *S. symbiotica* SCc is adapted to compensate for the losses in *B. aphidicola* BCc, and in conjunction functionally resemble a less genomically reduced *Buchnera*. And second, the sum of *Serratia* and *Buchnera* in *A. pisum* still clusters apart from the rest of *Serratia*, proof of the facultative state of *S. symbiotica* in the aphid *A. pisum* [12], failing to show a marked functional complementation with *Buchnera*. So, in the event of a facultative endosymbiont establishing a consortium with an already present obligate endosymbiont, like in the case of *S. symbiotica* SCc, the new consortium would be compelled to maintain general functionality of the previously present and well-established bacterium.

### Single-copy Core Genome Phylogeny

**Figure 3:**
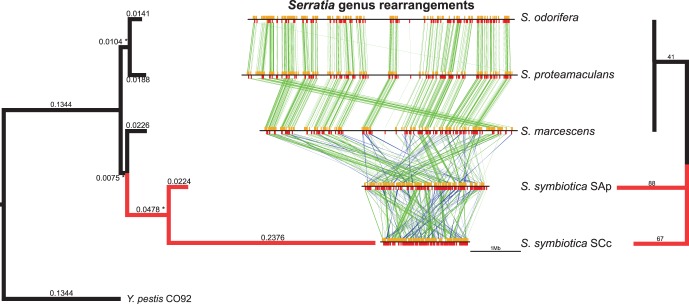
Phylogenetic and rearrangements history of the single-copy core genes of the *Serratia* spp. On the left side, rooted ML tree with * indicating bootstrap support values of 100 (percent of total). On the middle, pairwise synteny plots of free-living *S. marcescens*, *S. odorifera* and *S. proteamaculans* along with endosymbiotic relatives *S. symbiotica* SCc and *S. symbiotica* SAp. On the right side, unrooted minimal number of rearrangements tree as calculated by MGR. On red, branches from the endosymbiotic lineages.

It was of great importance to determine the phylogenetic position of both *S. symbiotica* among the *Serratia* in a maximum likelihood (ML) phylogenetic tree. In a past phylogenetic reconstruction by Burke *et al*. [12], they were unable to resolve with complete confidence the position of *S. symbiotica* SAp within the *Serratia* using various **γ**-proteobacteria. To approach this problem we chose the 580 single-copy genes of the *Serratia* spp. core genome that were shared with *Y. pestis* (used as an outgroup) and reconstructed a concatenated protein sequence phylogeny (**Figure 3:** **left**). A striking feature is the evident acceleration in the branch leading to *S. symbiotica* SCc in contrast to what is seen in the other *Serratia*, including *S. symbiotica* SAp. However, *S. symbiotica* SAp clusters with *S. symbiotica* SCc forming a symbiotic clade. It is worth mentioning that both *S. symbiotica* cluster with *S. marcescens*, which is the only one isolated from an insect (*D. melanogaster*) [37] from the strains used in this study, insinuating that the symbiotic lineage may have come from an insect-pathogen rather than a plant-pathogen *Serratia*.

### Rearrangements Across *Serratia*

Another interesting feature in the genomic evolution of endosymbionts is the invasion by mobile genetic elements. These elements can cause a high degree of rearrangements in the bacterial genomes undergoing adaptation to intracellular life [12], [19]. These elements are especially present in recent associations but lacking in ancient ones. For example, in the ancient and obligate endosymbionts *B. aphidicola* and *Blochmannia*, an extreme genome stasis has been described [3], [50] having a parallel evolution with its hosts. This contrasting what is seen in more recent associations like in the case of SOPE (*Sitophilus oryzae* primary endosymbiont) [19], facultative endosymbionts like *Sodallis glossinidius* [*20*], *Hamiltonella defensa* [10] and *Regiella insecticola* [11] or the recently sequenced genome of REIS (the *Rickettsia* endosymbiont of *Ixodes scapularis*) [21].

To study the rearrangements undergone by both *S. symbiotica* we decided to analyze the rearrangements of the single-copy core genes (**Figure 3:** **middle**). We can clearly see that among FLS, the synteny of the single-copy core is perfectly conserved among the strains, with the 597 single-copy genes being in the same order and orientation, except in the case of *S. marcescens* where the replication origin seems to be misplaced as checked by originX [51] (data not shown). This lets us assume these genes are present in the same order among *Serratia*, and thus we can assume that any reordering witnessed in *S. symbiotica* strains could be due to the invasion and/or mobilization of mobile genetic elements that occurred during the endosymbiotic genomic reduction [19]. In the case of both *S. symbiotica*, the level at which they have undergone genetic rearrangements becomes evident, even showing great rearrangements between the two. This means that the divergence of these two endosymbionts must have been prior to the loss of *S. symbiotica* SCc’s capacity to rearrange its genome.

We then calculated a minimal rearrangement phylogeny for the selected *Serratia* genomes (**Figure 3:** **right**). This method allows us to calculate a tree with the minimal number of rearrangements required to obtain an ancestral gene order. Strikingly, the minimal rearrangement distance from *S. symbiotica* SAp to FLS (129) is greater than that of *S. symbiotica* SCc to FLS (108), and the distance between them to a common ancestor (155) is the greatest. The rearrangements undergone in the recent endosymbiotic lineages might have happened in a random fashion due to the high numbers of mobile genetic elements and could also be facilitated by the relaxation in pressure of gene order in certain genes because of the degradation of the transcription regulation. This means that in different events of infection by *Serratia* endosymbionts or in early divergences, we might have very different gene orders.

### Conclusions

The study of this type of endosymbiotic organisms is shedding light on the differences between a free-living and obligate endosymbiotic state. Knowledge is also provided on adaptations to a nutrient-rich and stable environment, in which the bacteria cells undergo drastic changes in their genomes.

In the present study, we have found multiple evidences supporting the existence of two very distinct *S. symbiotica* lineages. One of which has obligate endosymbiotic characteristics (*S. symbiotica* SCc), such as accelerated evolution, with the COG profile being similar to that of the core genome, with a lack of mobile elements, no genetic redundancy and loss of almost all strain-specific genes. And a second one (*S. symbiotica* SAp), presenting all the traits of a facultative endosymbiont, with its functional profile being “intermediate” between that of *S. symbiotica* SCc and FLS, with the presence of mobile genetic elements and preserving still a great amount of strain-specific genes. In the case of the *Serratia* genus, commonly found in a variety of insects, it would not be surprising to find more endosymbiotic strains in different stages of lifestyle transition from FLS to more ancient and well-established obligate endosymbionts, as also proposed for *Wolbachia* [52] and *Ricketsia* [53], [54]. We were also able to determine the phylogenetic relationship among the different *Serratia* and place the symbiotic lineage closer to an insect-isolated strain indicating its probable insect-pathogen origin.

Even though we have gained insight into how the genetic rearrangements are happening, in order to have a better understanding of this process as well as the genetic decay and the transition from a free-living bacteria to an endosymbiotic one, more *S. symbiotica* must be analyzed to determine the basis and reason for the associations these bacteria have with aphids and its obligate endosymbiont *B. aphidicola*.

## Materials and Methods

### Construction of *Serratia* spp. Pan-genome

*Serratia* genomes were recovered from their respective databases (See **File S2**: **Table S1**), gene annotation from prediction of *S. marcescens* Db11 was done using **BASys** [55]. The protein sequences were fed into **OrthoMCL** [56] with an inflation value of 1.5, a 70% match cut-off, and e value cut-off of 1e-5. A total of 17, 086 coding genes were clustered into 4, 469 families of orthologous genes, leaving 2, 293 as single family genes. Clustering was checked for consistency using COG categories [45] to assess the homogeneity of COG assignment for all the genes in a given family, screening of clusters to check for inflation value cluster fragmentation effect, and gene number per family to make sure not many gene rich families arose. Visual display of the pan-genome subspaces was done using the **R** custom modified drawVennDiagram function of package gplots [57].

### COG Profiles

COGs categories were assigned using a series of Perl scripts to find non-overlapping hits against the COG database using **Blastp** with an e-value cut-off of 1e-03 [58]. The COG profile displays and clustering were made using the heatmap2 function from the **R** package gplots. This heatmap would represent a two way clustering having the most similar columns closer together and the most similar rows in the same fashion, showing the dissimilarity distances of columns with the top dendogram and the dissimilarity distance from the COG categories in the left one. Row reordering was chosen for the function for visual and categorization purposes. For assessing *S. symbiotica* divergence from FLS, absolute COG category frequencies were divided by the strains total number of COG assigned CDSs (see **File S1**: **Table S2**) and then subtracted the mean relative frequency from the FLS in the same COG category. Kruskal-Wallis tests were carried out on the absolute frequency tables of COG profiles using **R**. The categorization of retained/reduced COG categories in comparison to FLS (using the relative values table, as described above) was done in the following way: Extremely retained, more than 5% difference above zero; highly retained, more than 2 and less than 5% difference above zero; moderately retained, more than 0 and less than 2% difference above zero; moderately reduced, less than 0 and more than 2% difference below zero; highly reduced, less than 2 and more than 5% difference below zero; extremely reduced, less than 5% difference below zero. Important is to remark that even a lower than 1% difference is important since FLSs differences range between 0.0006% and 0.4253% with a mean of 0.1218% (visually displayed in **Figure 2A** by showing cells of the FLS in close-to-white tones).

### Phylogenetic Analysis

The 580 single-copy shared genes identified between *Serratia* spp. and *Yersinia pestis* CO92 were extracted and translated to amino-acid sequences using **transeq** from the EMBOSS suite [59] and aligned using the L-INS-i algorithm from **MAFFT** v6.717b [60] (See **file S2**). **Gblocks** [61] was used to refine the alignment. ML tree was calculated with 1000 bootstrap replicates using **RAxML** v7.2.6 [62]. Visual display of both trees was done using **FigTree** v1.3.1 and edited in **Inkscape**.

### Genome Rearrangements

In all, 597 single-copy genes (the “single-copy core”) were selected to study the rearrangement history of *Serratia* genus. Scaffold or contig order for unfinished genomes was determined with **MUMers** promer v3.22 [63] using as reference the genome of *S. proteamaculans* 568. Custom **Perl** scripts were developed to create input files for genome rearrangements plotting using **genoPlotR** v0.7 [64]. Minimal number of rearrangements phylogeny was calculated using **MGR** v2.03 [65] with the circular genomes option and without using any heuristics.

## Supporting Information

Figure S1 **Heatmap of COG profile clustering for selected** ***B. aphidicola*** **and** ***B. aphidicola*** **BCc plus** ***S.*** ** ***symbiotica*** **SCc functional profile.** Heatmap displaying the clustering of various *B. aphidicola* genomes along with the sum of the functional profiles for *B. aphidicola* BCc and its symbiotic partner *S. symbiotica* SCc, showing a closer clustering of these joint genomes to that of less genomically reduced *B. aphidicola* genomes. **BAp**: *B. aphidicola* from *A. pisum*; **BBp**: *B. aphidicola* from *B. pistaciae*; **BCc**: *B. aphidicola* from *C. cedri*; **BSg**: *B. aphidicola* from *S. graminum*; **SCc**: *S. symbiotica* from *C. cedri*.
(EPS)

File S1 **Supplementary tables.**
(DOC)

File S2 **Single-copy core genes shared with** ***Y. pestis*** **CO92 concatenated in FASTA format.**
(FAA)

Table S1 Relative values for each of COG category from the selected *Serratia* genomes.
(DOCX)

Table S2 Strains and accession numbers or sources for genomes used in this work.
(DOCX)
