# Supplementary material for: Comparative Genomics of Serratia spp.: Two Paths towards Endosymbiotic Life
Source: PLoS One. 2012 Oct 15;7(10):e47274. doi: 10.1371/journal.pone.0047274 (PMC3471834; doi:10.1371/journal.pone.0047274)
Supplement: File S1 — Supplementary tables. (DOC) [file pone.0047274.s002.doc]

### Supplementary tables

**Table S1.** Strains and accession numbers or sources for genomes used in this work.

| **Organism** | **Accession numbers or sources** | **Organism** | **Accession numbers or sources** |
| --- | --- | --- | --- |
| ***B. aphidicola* (BAp) 5A** | CP001161 | ***S. marcescens* Db11** | http://www.sanger.ac.uk/resources/downloads/bacteria/serratia-marcescens.html |
| ***B. aphidicola* (BAp) APS** | BA000003 | ***S. odorifera* 4Rx13** | ADBX00000000 |
| ***B. aphidicola* (BAp) LSR1** | ACFK00000000 | ***S. proteamaculans* 568** | CP000826 |
| ***B. aphidicola* (BAp) Tuc7** | CP001158 | ***S. symbiotica* SAp** | AENX00000000 |
| ***B. aphidicola* (BBp)** | AE016826 | ***S. symbiotica* SCc** | CP002295.1 |
| ***B. aphidicola* (BCc)** | CP000263 | ***Y. pestis CO92*** | AL590842.1 |
| ***B. aphidicola* (BSg)** | AE013218 |  |  |

**Table S2.** Relative values for each of COG category from the selected *Serratia* genomes.

| **COG** | **Pangenome** | **Core** | **Smar** | **Sodo** | **SsCc** | **Spro** | **SsAp** |
| --- | --- | --- | --- | --- | --- | --- | --- |
| **A** | 0,00034 | 0,00162 | 0,00025 | 0,00024 | 0,00149 | 0,00023 | 0,00064 |
| **B** | 0,00020 | 0,00000 | 0,00025 | 0,00024 | 0,00000 | 0,00023 | 0,00000 |
| **C** | 0,06070 | 0,05340 | 0,06040 | 0,06387 | 0,04903 | 0,06272 | 0,05246 |
| **D** | 0,01036 | 0,02265 | 0,00884 | 0,00858 | 0,02080 | 0,00846 | 0,01983 |
| **E** | 0,09931 | 0,05178 | 0,10410 | 0,10748 | 0,05498 | 0,10524 | 0,06782 |
| **F** | 0,02561 | 0,05016 | 0,02234 | 0,02407 | 0,04755 | 0,02373 | 0,03391 |
| **G** | 0,08894 | 0,05340 | 0,09158 | 0,09867 | 0,05498 | 0,09725 | 0,04798 |
| **H** | 0,03827 | 0,06634 | 0,03388 | 0,03551 | 0,07281 | 0,03242 | 0,05822 |
| **I** | 0,03360 | 0,04854 | 0,03413 | 0,03408 | 0,04458 | 0,03453 | 0,02367 |
| **J** | 0,05521 | 0,18285 | 0,04346 | 0,04409 | 0,18128 | 0,04369 | 0,09277 |
| **K** | 0,09714 | 0,03398 | 0,10680 | 0,10867 | 0,02972 | 0,10430 | 0,05054 |
| **L** | 0,04525 | 0,05987 | 0,03535 | 0,03384 | 0,06686 | 0,04346 | 0,09725 |
| **M** | 0,06435 | 0,10356 | 0,06285 | 0,06339 | 0,10253 | 0,05732 | 0,07358 |
| **N** | 0,02249 | 0,00485 | 0,02701 | 0,02026 | 0,00446 | 0,02607 | 0,01472 |
| **O** | 0,03739 | 0,06311 | 0,03609 | 0,03456 | 0,05795 | 0,03430 | 0,04798 |
| **P** | 0,05927 | 0,03883 | 0,06384 | 0,06220 | 0,04606 | 0,05826 | 0,04798 |
| **Q** | 0,01890 | 0,01294 | 0,02013 | 0,02145 | 0,01189 | 0,01903 | 0,01152 |
| **R** | 0,09782 | 0,06472 | 0,10066 | 0,09795 | 0,06241 | 0,10054 | 0,09789 |
| **S** | 0,08922 | 0,04369 | 0,09354 | 0,08651 | 0,04606 | 0,09255 | 0,09469 |
| **T** | 0,03096 | 0,00971 | 0,03388 | 0,03122 | 0,01189 | 0,03242 | 0,02687 |
| **U** | 0,01253 | 0,02589 | 0,00810 | 0,01120 | 0,02377 | 0,01081 | 0,02751 |
| **V** | 0,01213 | 0,00809 | 0,01252 | 0,01192 | 0,00892 | 0,01245 | 0,01216 |
