# Supplementary material for: Comparative Genomics of Serratia spp.: Two Paths towards Endosymbiotic Life
Source: PLoS One. 2012 Oct 15;7(10):e47274. doi: 10.1371/journal.pone.0047274 (PMC3471834; doi:10.1371/journal.pone.0047274)
Supplement: Table S1 — Relative values for each of COG category from the selected Serratia genomes. (DOCX) [file pone.0047274.s004.docx]

**Table S1.** Strains and accession numbers or sources for genomes used in this work.

| **Organism** | **Accession numbers or sources** | **Organism** | **Accession numbers or sources** |
| --- | --- | --- | --- |
| ***B. aphidicola* (BAp) 5A** | CP001161 | ***S. marcescens* Db11** | http://www.sanger.ac.uk/resources/downloads/bacteria/serratia-marcescens.html |
| ***B. aphidicola* (BAp) APS** | BA000003 | ***S. odorifera* 4Rx13** | ADBX00000000 |
| ***B. aphidicola* (BAp) LSR1** | ACFK00000000 | ***S. proteamaculans* 568** | CP000826 |
| ***B. aphidicola* (BAp) Tuc7** | CP001158 | ***S. symbiotica* SAp** | AENX00000000 |
| ***B. aphidicola* (BBp)** | AE016826 | ***S. symbiotica* SCc** | CP002295.1 |
| ***B. aphidicola* (BCc)** | CP000263 | ***Y. pestis CO92*** | AL590842.1 |
| ***B. aphidicola* (BSg)** | AE013218 |  |  |
